# Content-Free Awareness: EEG-fcMRI Correlates of Consciousness *as Such* in an Expert Meditator

**DOI:** 10.3389/fpsyg.2019.03064

**Published:** 2020-02-18

**Authors:** Ulf Winter, Pierre LeVan, Tilmann L. Borghardt, Burak Akin, Marc Wittmann, Yeshe Leyens, Stefan Schmidt

**Affiliations:** ^1^Department of Psychosomatic Medicine and Psychotherapy, Medical Center – University of Freiburg, Faculty of Medicine, University of Freiburg, Freiburg im Breisgau, Germany; ^2^Department of Radiology – Medical Physics, Medical Center – University of Freiburg, Faculty of Medicine, University of Freiburg, Freiburg im Breisgau, Germany; ^3^Departments of Radiology and Paediatrics, Hotchkiss Brain Institute and Alberta Children’s Hospital Research Institute, University of Calgary, Calgary, AB, Canada; ^4^The Ekayana Institute, Lenzkirch, Germany; ^5^Center for Basics in NeuroModulation (NeuroModulBasics), Faculty of Medicine, University of Freiburg, Freiburg im Breisgau, Germany; ^6^Institute for Frontier Areas of Psychology and Mental Health, Freiburg im Breisgau, Germany

**Keywords:** content-free awareness (CFA), consciousness as such, neural correlate of consciousness (NCC), disconnected consciousness, meditation, default-mode network (DMN), dorsal attention network (DAN), EEG-fMRI

## Abstract

The minimal neural correlate of the conscious state, regardless of the neural activity correlated with the ever-changing contents of experience, has still not been identified. Different attempts have been made, mainly by comparing the normal waking state to seemingly unconscious states, such as deep sleep or general anesthesia. A more direct approach would be the neuroscientific investigation of conscious states that are experienced as free of any specific phenomenal content. Here we present serendipitous data on content-free awareness (CFA) during an EEG-fMRI assessment reported by an extraordinarily qualified meditator with over 50,000 h of practice. We focused on two specific cortical networks related to external and internal awareness, i.e., the dorsal attention network (DAN) and the default mode network (DMN), to explore the neural correlates of this experience. The combination of high-resolution EEG and ultrafast fMRI enabled us to analyze the dynamic aspects of fMRI connectivity informed by EEG power analysis. The neural correlates of CFA were characterized by a sharp decrease in alpha power and an increase in theta power as well as increases in functional connectivity in the DAN and decreases in the posterior DMN. We interpret these findings as correlates of a top-down-initiated attentional state excluding external sensory stimuli and internal mentation from conscious experience. We conclude that the investigation of states of CFA could provide valuable input for new methodological and conceptual approaches in the search for the minimal neural correlate of consciousness.

## Introduction

Neuroscientific meditation research has risen sharply during the last decade, thereby initiating the new field of contemplative neuroscience. So far the majority of studies in this field have explored the physiological mechanisms of meditation, changes in brain structure and function associated with meditation practice, as well as differences in cognitive abilities between meditators and non-meditators (Davis and Vago, 2013; Vieten et al., 2018). Only few studies have investigated deep states of meditation and extraordinary experiences, such as ‘enlightenment’ or ‘awakening.’ This is understandable, considering the multiplicity of methodological obstacles and the elusive nature of such experiences that, however, occur more frequently than expected and are usually evaluated as extremely meaningful or even life-changing by the meditators (Davis and Vago, 2013).

The investigation of deep states of meditation could open a unique pathway in identifying the neural correlates of consciousness, complementing approaches investigating seemingly unconscious states in deep sleep, general anesthesia or disorders of consciousness (Winter, 2013). Meditative states of consciousness without any specific content are described in all major contemplative traditions. Whether such states of consciousness actually exist is the subject of current debates in the philosophy of mind and the Neurosciences (Gennaro, 2008; Millière et al., 2018; Winter, 2020).

A state of ‘content-free awareness’ (CFA) represents a non-pathological, wakeful state of consciousness free of the complexity of normal wakeful states, i.e., the bare state of being aware or consciousness *as such*.^1^ The neural correlate of such a minimal form of conscious experience may represent the minimal neural system sufficient for conscious experience (Chalmers, 2000), i.e., the *minimal* neural correlate of consciousness (Winter, 2013, 2020).

We are presently investigating the neurophysiologic correlates of meditation-induced states of awareness with reduced content in expert and novice meditators from different traditions by applying the latest technical developments in EEG-fMRI technology. Here we present serendipitous data consisting of an EEG-fMRI measurement in an extraordinarily qualified meditator who reported having had an experience of ‘awakening’ during his meditation in the scanner. The former Buddhist monk described his experience as a ‘clear, aware openness’ without any thoughts, physical, or sensory perception and even without any sense of self, time, and space – a state traditionally called the ‘ground state of awakening.’ His experience appears to be a case of bare CFA.

The experience of selfless and objectless awareness occurs only in a small number of meditators and usually only after years of practice (Wittmann, 2018). It is described as an experience that cannot be controlled by the meditator. We were very fortunate to have been able to record such an experience under the extremely unfavorable external conditions of an MRI examination. Therefore, despite the limitations of a single-case study, we decided to publish this data in advance in a brief research report.

To explore the neural correlates of this experience, we focused on two specific cortical networks related to external and internal awareness, i.e., the dorsal attention network (DAN) and the default mode network (DMN), respectively. Changes in activity and connectivity within and between these two networks seem to play a pivotal role for the loss of consciousness in sleep (Sämann et al., 2011), anesthesia (Schrouff et al., 2011) and disorders of consciousness (Boly et al., 2012), as well as in altered states of consciousness, such as hypnosis (Demertzi et al., 2011), meditation (Brewer et al., 2011; Josipovic et al., 2012), and psychedelic experiences (Carhart-Harris et al., 2014). To investigate the neural correlates of the reported disconnection of conscious experience from external sensory stimuli, we also analyzed the interplay of DAN with the primary auditory cortex (PAC).

We analyzed the dynamic aspects of fMRI connectivity, further informed by EEG spectral analysis. For this we used EEG spectral power in the two frequency bands most commonly associated with meditation: theta and alpha (Cahn and Polich, 2006; Lomas et al., 2015). EEG alpha power is a marker for cognitive and attentional processes (Klimesch, 1999; Katyal et al., 2019) and specifically associated with DMN (Jann et al., 2009) and DAN (Hacker et al., 2017). Alpha oscillations may play a crucial role for the attentional and cognitive control processes involved in meditation (Marzetti et al., 2014; Raffone and Srinivasan, 2017).

Similar to alpha power, theta power was found to be associated with cognitive control (Başar et al., 2001; Cavanagh and Frank, 2014) and attention (Keller et al., 2017). Increases in frontal midline theta power have been found across a variety of meditation practices (Cahn and Polich, 2006; Lomas et al., 2015) and especially during concentrative meditation (Baijal and Srinivasan, 2010; Aftanas and Golocheikine, 2014). These findings identify frontal midline theta power as “a key contributor to meditative neural dynamics” (Cahn et al., 2010, p. 49). Similar to alpha, specific associations with the DMN BOLD activity were found for theta oscillations (White et al., 2013; Hacker et al., 2017).

Based on the previous research (see above), we expected changed and basically decreased functional connectivity within the DMN and the DAN, decreased anticorrelation between this two networks as well as decreased connectivity between the DAN and the PAC during CFA. We also expected increases in theta and alpha power and signs of deep relaxation in the heart-rate and breathing data.

## Methods

### Participant

The expert meditator (TB; 56 years old) is a former physician and now a qualified teacher in the Tibetan Buddhist Karma Kagyü tradition. TB has been meditating for 40 years, lived as an ordained monk in a retreat center for 21 years, and was a teacher there for 17 years. He meditated for up to 12 h a day for 10 years and has spent an estimated total of over 50,000 h in formal meditative practice. TB’s exceptional introspective skills have already helped us to elucidate the experiential nature of other neurophysiological signals (Schmidt et al., 2016).

### Procedure

Recording took place in the Department of Radiology at the University Medical Center in Freiburg, Germany. The recording was divided into five scanning runs during which the participant was instructed to keep his eyes closed. A 5-min baseline measure was first performed during which TB was instructed to remain relaxed and alert without engaging in any formal meditation practice. During the runs 2, 3, and 4 (each 3 min in length) TB was asked to focus on external sensory perceptions (i.e., the sound of the scanner), internal mentation (i.e., an episodic memory exercise) and bodily sensations, respectively. Finally, TB was instructed to engage in formal meditation to reach a state of content-minimized awareness (30 min). Between each run TB provided self-ratings regarding mentation, the outer senses, bodily self-awareness, and the experience of space and time during the previous condition using a visual 10-step rating scale controlled by a touchpad. TB was interviewed in detail about his experience after completion of the recording.

#### Data Acquisition

Neurophysiologic data was acquired by simultaneous EEG and fMRI recordings. The EEG was recorded using a MR-compatible, high-resolution, 256-channel surface EEG system (Geodesic EEG System 410, Electrical Geodesics, Eugene, OR, United States) with a bandpass filter between 0.1 and 400 Hz and a sampling rate of 1000 Hz. The fMRI was acquired on a Siemens 3T Prisma scanner (Siemens Healthineers, Erlangen, Germany) using the following parameters: magnetic resonance encephalography (MREG) sequence (Assländer et al., 2013), TR = 100 ms, TE = 36 ms, flip angle 25°, 3D field of view 192 mm × 192 mm × 150 mm, and a spatial resolution of 3 mm × 3 mm × 3 mm. The ECG and respiration data were recorded using the physiological monitoring unit of the MR scanner.

#### Data Analysis

**FIGURE 1:**
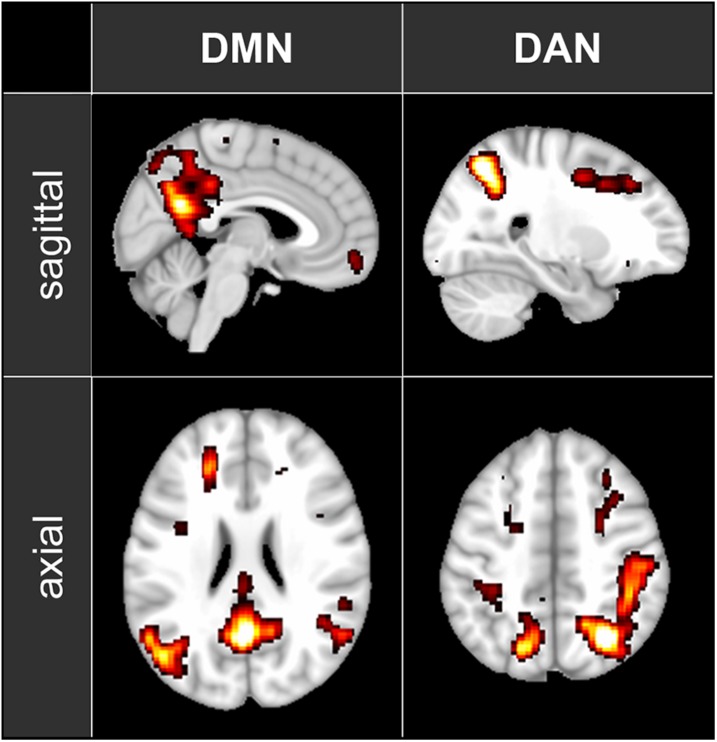
The maps of the two ICA components representing DMN and DAN that have been used in the functional connectivity analysis. The maps are shown in axial and sagittal views superimposed on a standard MNI152 T1 2 mm resolution brain template. The following areas are visible: DMN: posterior cingulate cortex (with the adjacent regions of the precuneus), left and right inferior parietal lobule and medial prefrontal cortex; DAN: left and right intraparietal sulcus, left and right frontal eye field.

fMRI data were pre-processed using FSL (FMRIB’s Software Library^2^). Images were motion-corrected, transformed into standard MNI space and smoothed with a 6-mm FWHM Gaussian kernel. To localize the DMN, the DAN and the PAC, the 5-min baseline segment was decomposed into 100 independent spatial components by probabilistic independent component analysis (ICA) as implemented in MELODIC Version 3.14, part of FSL (Beckmann and Smith, 2004). The 100 resulting spatial maps were visually inspected to identify the components corresponding to DMN and DAN (see Figure 1) as well as the left and right PAC. For each analyzed condition, the fMRI time series were segmented into 60-s, non-overlapping time windows. Functional connectivity within each time window was then computed as Fisher-transformed correlation coefficients between each pair of nodes belonging to the previously identified independent component maps.

EEG data were pre-processed and analyzed using in-house MATLAB scripts and Brain Vision Analyzer (Brain Products, Gilching, Germany). Gradient and pulse artifacts were corrected by previously described in-house approaches (LeVan et al., 2013, 2016). Bandpass (0.5 to 70 Hz) and notch filters (48–52 Hz) were applied to correct for potential line noise. The data were divided in 60-s segments, re-referenced to average, and segmented into 2-s epochs. For each 60-s segment, at least 25 artifact-free epochs were transformed into the frequency domain using the fast-Fourier-transform (FFT) and averaged. The absolute spectral power at each of the 60 electrodes of the extended 10–20 system was computed for the theta (4–8 Hz) and alpha (8–12 Hz) frequency bands.

ECG data were analyzed for heart rate (HR) and heart-rate variability (HRV) using Kubios HRV Standard Software 2.0 (Kuopio, Finland). Successive R-peaks (R–R intervals) in the ECG signal were extracted, artifact corrected, and detrended using the smoothness-priors approach. Based on these R–R intervals we computed the root mean square of successive differences (RMSSD), a time-domain indicator for the HRV reported to be largely independent of respiratory effects (Hill and Siebenbrock, 2009). Respiration data were used to trace the breathing rate (BR) and to calculate the estimated oxygen consumption (OCe) as tidal volume multiplied by BR.

#### Data Editing

Only the resting-state baseline and the meditation condition were evaluated in this single-case study. According to TB’s report (see below), we decided to consider the last 5 min of the meditation condition as representative of CFA. In contrast, we consider the first 25 min of the meditation condition as a content-related meditative state, which was not the focus of our analysis. We used the 5-min segment in the middle of these 25-min meditation epoch (i.e., min 11 to 15) as ‘content-related meditation’ (CRM) for additional analyses. The data of the complete 30-min meditation condition were only used to trace the time courses of the various measurements and to calculate the correlation between the time courses of fMRI connectivity and EEG spectral power.

### Statistical Analyses

For all measures we analyzed the values in segments of 60 s in length for the 5-min baseline and for the 30-min meditation condition. To identify significant differences between all three conditions (CFA, CRM and rest) we applied a one-way ANOVA with contrasts.

Furthermore, we investigated a possible association between changes in fMRI connectivity and changes in the EEG spectral power. For this purpose, we correlated the connectivity time courses of those pairs of network nodes which showed significant connectivity differences for the contrast CFA vs. rest, with the time courses of EEG theta and alpha power.

To account for potentially independent EEG power changes in different regions we computed the spectral power at those electrodes, which showed the highest relative power differences between CFA and rest, namely F6, F7, and Pz for the theta band and FCz, P7, P8 for the alpha band. The correlations between fMRI connectivity and EEG alpha power were calculated and tested over the full 35 min of the baseline plus the meditation condition. The statistical threshold for all tests was set at *p* = 0.05. We applied FDR-correction in all analyses.

## Results

### Subjective Report

The subjective ratings during the session show very clear differences in the amount of consciously experienced content. For the baseline condition, TB rated the total amount of mental content (sensory perceptions, thoughts, and mental images) at 8 on the 10-point scale. The intensity of bodily self-awareness and the experience of time and space were rated at 8 and 9, respectively. In contrast, for the 30-min meditation condition as a whole, the same three items were rated at 2 (sensory perceptions and imaginings), 2 (bodily self-awareness), and 1 (experience of time and space). In the interview after the session, TB declared that during the meditation condition the meditative state gradually deepened and reached the deepest level during the last few minutes of this period. In this last phase, he reported that he had no awareness of any mental content or any sensory event, including the noise of the MRI scanner. Similarly, he reported having had no experience of self, time, or space of any kind whatsoever at this stage. He was sure he had been awake the entire time, and the EEG data showed no sleep-related signs.

He also reported feeling deeply refreshed after this condition, which he recognizes from earlier experience as a sign of reaching a very deep state of ‘non-dual’ consciousness (i.e., without subject-object perception). TB appraised this experience – in terms of Tibetan Buddhism – as the experience of basic *mahāmudrā*, i.e., absolute openness without any activation of the senses.

### ECG and Respiration

**FIGURE 2:**
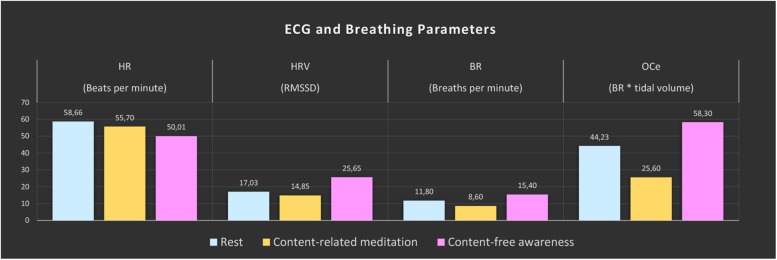
ECG and breathing measures. The color bars represent the median values of the 5-min epochs of rest (blue), content-related meditation (yellow) and content-free awareness (pink) in each case. From left to right: heart rate (HR), heart-rate variability (HRV, RMSSD = root mean square of successive differences), breathing rate (BR), estimated oxygen consumption (OCe).

Large changes were found for all computed ECG and respiration measures during CFA compared to the resting-state baseline as well as compared to CRM (see Figure 2). During the meditation condition HR gradually dropped from the baseline level and reached the lowest values during the CFA phase. HRV (RMSSD), BR, and OCe were well below the baseline level during most of the CRM phase, but started to increase after about 20 min and reached values significantly above baseline level during the CFA phase. The differences between CFA and the resting state as well as between CFA and CRM were significant for all measured values. For the contrast CRM vs. rest, only the decreases in HR and OCe were significant (see Table 1).

**TABLE 1:** Significance test results of the ECG and breathing parameters. Shown are the results of a one-way ANOVA with contrasts between the three conditions rest, content-related meditation (CRM) and content-free awareness (CFA).

ECG/breathing parameters		CFA vs. Rest	CFA vs. CRM	CRM vs. Rest
HR	*t*	−15.493	−10.181	−5.311
	*df*	12	12	12
	*p (FDR)*	< 0.001	< 0.001	< 0.001
HRV	*t*	5.280	6.615	−1.335
	*df*	12	12	12
	*p (FDR)*	< 0.001	< 0.001	*ns* (0.21)
BR	*t*	6.573	5.919	−2.807
	*df*	12	5.083	4.952
	*p (FDR)*	< 0.001	0.002	*ns* (0.051)
OCe	*t*	2.841	6.603	−3.762
	*df*	12	12	12
	*p (FDR)*	0.015	< 0.001	< 0.01

*HR = Heart rate, HRV = Heart rate variability, BR = Breathing rate, OCe = Estimated oxygen consumption, *p* (FDR) = False discovery rate corrected *p*-value, ns = non-significant.*

### EEG Spectral Power

The analysis of spectral power in the theta (4–8 Hz) and alpha (8–12 Hz) frequency bands demonstrated clear differences between the three analyzed conditions.

**FIGURE 3:**
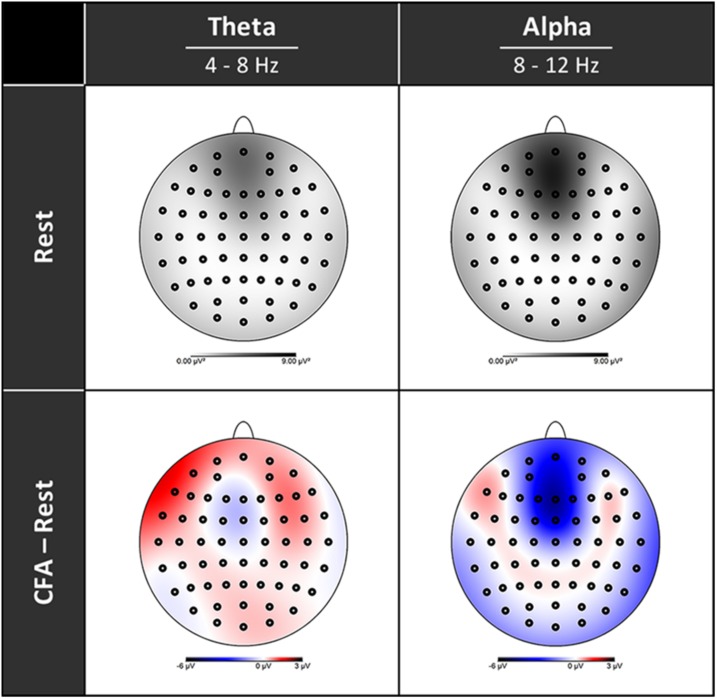
Topographies of EEG spectral power. Upper row rest condition, lower row difference between content-free awareness (CFA) and rest. Increases in power are displayed in red, decreases in blue. Black dots denote the electrodes of the extended 10–20 system.

**FIGURE 4:**
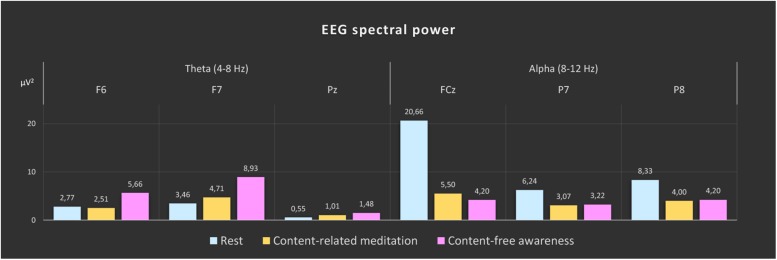
EEG spectral power during rest, content-related meditation and content-free awareness. For each frequency band, the electrodes with the most significant differences for the main contrast (content-free awareness vs. rest) are shown (theta band: F6, F7, and Pz; alpha band: FCz, P7, and P8). The significance values for the different contrasts are given in Table 2. The color bars represent the median values of spectral power (μV^2^) of the 5-min epochs of rest (blue), content-related meditation (yellow) and content-free awareness (pink) in each case.

In Figure 3 the topographies during the resting state and the increases and decreases during CFA compared to rest are presented. Figure 4 shows theta and alpha power for those three electrodes having demonstrated the highest relative difference between CFA and rest in each case. The statistical details are listed in Table 2. Noteworthy is the unusual topography of the alpha power during the resting-state condition. While in most people alpha activity has a clear occipital dominance, TB showed a striking frontal dominance and little occipital activity (see Figure 3). For the main contrast CFA vs. rest, both the increases in theta power (at F6, F7, and Pz) as well as the decreases in alpha power (at FCz, P7, and P8) were significant at *p* < 0.001. The theta power showed a strong increase in frontopolar, lateral prefrontal and frontocentral as well as medial parietooccipital regions, especially during CFA compared to CRM (*p* = 0.001 at F6, F7, and Pz). During CRM compared to rest only the parietal theta power increase (at Pz) was significant (*p* < 0.001). In contrast, the alpha power dropped dramatically in the medial prefrontal and frontocentral area as well as in peripheral temporal and parietooccipital regions, already during CRM compared to rest (*p* = 0.001 at FCz, P7, and P8). During CFA compared to CRM only the frontal alpha power (at FCz) further decreased significantly (*p* < 0.05).

**TABLE 2:** Significance test results of the EEG spectral power in the theta (4–8 Hz) and alpha (8–12 Hz) frequency bands.

EEG spectral power		CFA vs. Rest	CFA vs. CRM	CRM vs. Rest
Theta (F6)	*t*	11.217	12.028	−0.811
	*df*	12	12	12
	*p (FDR)*	1.53E-07	2.83E-07	*ns* (0.43)
Theta (F7)	*t*	10.135	8.120	2.015
	*df*	12	12	12
	*p (FDR)*	3.72E-07	9.68E-06	*ns* (0.08)
Theta (Pz)	*t*	11.642	5.581	6.061
	*df*	12	12	12
	*p (FDR)*	1.35E-07	2.40E-04	8.50E-05
Alpha (FCz)	*t*	−22.480	−3.083	−20.311
	*df*	4.382	6.461	5.092
	*p (FDR)*	1.08E-05	0.029	9.06E-06
Alpha (P7)	*t*	−13.290	0.864	−14.154
	*df*	12	12	12
	*p (FDR)*	6.73E-08	*ns* (0.40)	2.45E-08
Alpha (P8)	*t*	−12.851	1.205	−14.056
	*df*	12	12	12
	*p (FDR)*	6.73E-08	*ns* (0.30)	2.45E-08

*Shown are the results of a one-way ANOVA with contrasts between the three conditions rest, content-related meditation (CRM) and content-free awareness (CFA). *p* (FDR) = False discovery rate corrected *p*-value, ns = non-significant.*

### fMRI Connectivity

**FIGURE 5:**
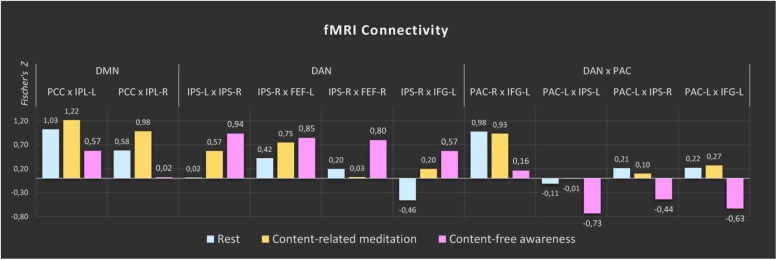
fMRI connectivity during rest, content-related meditation and content-free awareness. For each network, only the pairs of nodes with the most significant differences for the main contrast (content-free awareness vs. rest) are shown. A complete list of all significant differences is given in Table 3. The color bars represent the median values of Fisher-transformed correlation coefficients of the 5-min epochs of rest (blue), content-related meditation (yellow) and content-free awareness (pink) in each case. DMN = default mode network, DAN = dorsal attention network, PAC = primary auditory cortex, PCC = posterior cingulate cortex, IPL = inferior parietal lobule, IPS = intraparietal sulcus, FEF = frontal eye field, IFG = inferior frontal gyrus, L = left, R = right.

**FIGURE 6:**
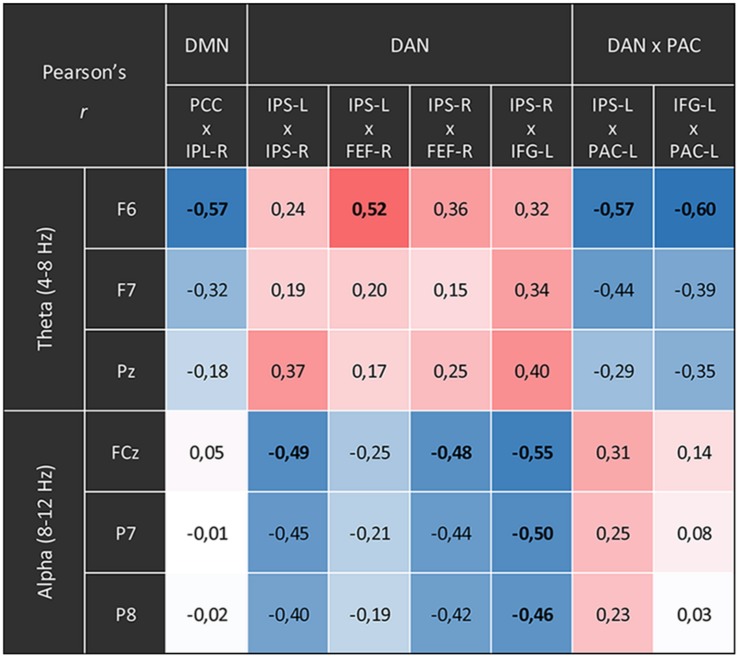
Correlations between the time courses of fMRI connectivity and EEG power. Shown are all Pearson’s correlation coefficients of time courses with significant differences for the main contrast (content-free awareness vs. rest). Blue cells display negative correlations red cells display positive ones. Significant correlations (*p* < 0.05, FDR-corrected) are printed in bold.

The comparisons between the three analyzed conditions (rest, CRM, CFA) revealed significant differences in BOLD functional connectivity within the DMN and the DAN as well as between DAN and the PAC (For details see Table 3). Figure 5 displays the connectivity values during each of the three analyzed conditions for the pairs of nodes that yielded the most significant differences for the main contrast of interest (CFA vs. rest). The correlations between the time courses of fMRI connectivity and EEG spectral power are shown in Figure 6.

**TABLE 3:** Significance test results of the fMRI connectivity. Shown are the results of a one-way ANOVA with contrasts between the three conditions rest, content-related meditation (CRM) and content-free awareness (CFA).

Network	Pair of nodes	*t*	*df*	*p*(FDR)
**CFA vs. Rest**				
DMN	PCC x IPL-L	−2.681	12	<0.05
	PCC x IPL-R	−3.983	12	<0.05
DAN	IPS-L x IPS-R	6.413	12	<0.01
	IPS-L x FEF-L	3.506	7.482	<0.05
	IPS-L x FEF-R	2.251	12	<0.05
	IPS-R x FEF-L	4.001	12	<0.01
	IPS-R x FEF-R	4.944	12	<0.01
	IPS-R x IFG-L	5.479	12	<0.001
DAN x PAC	PAC-R x IFG-L	−3.266	12	<0.01
	PAC-L x IPS-L	−3.371	12	<0.01
	PAC-L x IPS-R	−4.222	12	<0.01
	PAC-L x FEF-L	−2.717	12	<0.05
	PAC-L x IFG-L	−4.204	12	0.001
	PAC-L x IFG-R	−2.504	12	0.01
**CFA vs. CRM**				
DMN	PCC x MPFC	−3.331	12	<0.05
	PCC x IPL-L	−4.057	12	<0.05
	PCC x IPL-R	−7.325	12	<0.001
	IPL-L x IPL-R	−3.104	12	<0.05
DAN x PAC	PAC-R x FEF-R	−4.311	12	<0.01
	PAC-R x IFG-L	−3.110	12	<0.05
	PAC-R x IFG-R	−3.836	12	<0.01
	PAC-L x IPS-L	−3.280	12	<0.05
	PAC-L x IPS-R	−3.910	12	<0.01
	PAC-L x FEF-R	−2.993	12	<0.05
	PAC-L x IFG-L	−4.782	12	<0.01
	PAC-L x IFG-R	−3.181	12	<0.05
**CRM vs. Rest**				
DMN	PCC x IPL-R	4.191	8	<0.05
	IPL_L x IPL-R	4.476	8	<0.05
DAN	IPS-R x IFG-L	4.295	8	0.021

*DMN = default mode network, DAN = dorsal attention network, PAC = primary auditory cortex, PCC = posterior cingulate cortex, IPL = inferior parietal lobule, IPS = intraparietal sulcus, FEF = frontal eye field, IFG = inferior frontal gyrus, L = left, R = right, *p* (FDR) = False discovery rate corrected *p*-value.*

Compared to the resting state, CFA was characterized by widespread connectivity increases in DAN as well as decreased connectivity in posterior DMN and between DAN and left PAC. CFA compared to CRM was marked by widespread connectivity decreases in DMN and between DAN and bilateral PAC. During CRM compared to the resting state connectivity increased in the DAN and the posterior DMN.

The time courses of functional connectivity inside the right posterior DMN as well as of the connectivity between left-hemispheric DAN and PAC were negatively correlated with the time course of the right-frontal theta power. Besides, this right-frontal theta power also was positively correlated with connectivity of one pair of nodes inside the DAN. The connectivity in other parts of the DAN was negatively correlated with both frontal and parietal alpha power.

## Discussion

Our study explored the neural correlates of a meditative state, which, according to the retrospective report, was characterized by clear awareness without any kind of specific experiential content during the final phase. Consistent with the statements made in the interview, the information provided in the ratings during the session described a profound, strongly content-reduced experience. As expected, we found signs of parasympathetic activation in the ECG data, increases in EEG theta power as well as reduced functional connectivity in the DMN and between DAN and PAC during the CFA phase. We unexpectedly found an acceleration in the breathing rate, sharp decreases in EEG alpha power as well as increases in functional connectivity in the DAN and unchanged connectivity between DAN and DMN.

The physiological data indicate profound changes during the CFA phase compared to the resting state as well as compared to the CRM phase. The slowing of the heart rate and the increase in HRV (RMSSD) indicate a dominance of parasympathetic activity and deep physical relaxation. These results correlate with the findings of several studies on meditation and yoga (Krygier et al., 2013; Vinay et al., 2016). However, the increase in the respiratory rate and the oxygen consumption seems rather counterintuitive and more indicative of sympathetic activity. Traditional Buddhist concepts see meditation as a state of consciousness balanced between arousal and relaxation, avoiding the excesses of restlessness and drowsiness (Wallace, 2006). Arousal responses have been found before in meditators from the Hindu tantric yoga and Tibetan Buddhist traditions in particular, traditions that emphasize the ‘awake quality’ of the mind (Benson et al., 1990; Amihai and Kozhevnikov, 2014, 2015).

Our finding of a sharp decline in EEG alpha power deviates from the findings of the majority of previous studies on meditation, that yield an increase in alpha power, particularly in frontal areas, as the most common finding (Cahn and Polich, 2006; Lomas et al., 2015). However, in relation to these results it needs to be considered that it is not always useful to compare the characteristics of a specific conscious state of one participant to aggregated findings resulting from a meta-analysis.

Evidence exists suggesting that increases in alpha-power during meditation may be specific to meditation beginners and that experienced meditators may show decreases in alpha power rather than increases (Cahn et al., 2010). Decreased alpha-power levels were only found in studies that examined experienced meditators (Das and Gastaut, 1957; Baijal and Srinivasan, 2010; Lehmann et al., 2012; Hinterberger et al., 2014). As in the case of increased sympathetic activity mentioned above, the participants in these studies were largely from Hindu yoga and Tibetan Buddhist traditions.

We therefore assume that the meditation tradition and the extremely high level of experience of TB could play a role in our respiration and alpha-power findings. The unusual topography of the alpha power – with a frontal dominance – could be interpreted as a trait effect of long-term training. It should also be noted that the specific meditative state examined here is an exceptionally advanced state and is hardly comparable to the conditions studied in most meditation studies. Therefore, changes in alpha power found in states of consciousness disconnected from sensory stimuli (see below) may be more informative than the findings from meditation studies with less experienced meditators.

In general, higher alpha power has been found to be associated with an inhibition of the neural activity in the corresponding regions (Jensen and Mazaheri, 2010; Haegens et al., 2011). Changes in alpha power have been reported in fundamental top-down-modulated attentional processes such as spatial selection and distractor suppression (Kelly et al., 2006; Banerjee et al., 2011; Foxe and Snyder, 2011), as well as in working memory tasks (Palva et al., 2010; van Dijk et al., 2010). Reduced alpha power has also been found to be associated with higher attentiveness (Macdonald et al., 2011) and reduced mind-wandering (Baldwin et al., 2017).

Thus, our finding of reduced alpha power at frontal midline electrodes may correspond to a disinhibition and an increase in neural activity in structures related to cognitive control and attention. Decreases in alpha power have also been linked to states of diminished vigilance in drowsiness and sleep (Klimesch, 1999). However, our participant was awake during the whole session, and the EEG data show no sleep-related signs. Latest findings from research in REM sleep and ketamine anesthesia suggest that a decrease in alpha power may be a marker for a disconnection of consciousness from sensory stimuli (Sanders et al., 2012; Darracq et al., 2018; Comsa et al., 2019). Increases in theta power have been found to be associated with cognitive control and stable attention (Cavanagh and Frank, 2014; Keller et al., 2017) as well as with decreased activity in the DMN (White et al., 2013; Hacker et al., 2017), which is related to self-processing and mind-wandering.

We interpret our findings of a dramatically decreased alpha power and an increased theta power during content-reduced meditation (CRM) and CFA in two ways: on the one hand, it points to a considerably reduced self-referential mental activity. On the other hand, reduced alpha power may be an indication of a top-down-modulated disconnection of consciousness from sensory stimuli which closely resembles that initiated by bottom-up mechanisms in sleep and anesthesia. This interpretation is supported by the anti-correlation between the DAN and the PAC observed in the CFA phase, which may indicate auditory disconnection.

However, the substantially increased theta power too is probably of decisive importance here: while the alpha power decrease already takes place largely in the CRM and remains stable in the transition to CFA, the actual difference between CRM and CFA is reflected in the strong increase in theta power. In addition, the reduction of connectivity between the DAN and the PAC is negatively correlated with the frontal theta power increase. Therefore, the frontal theta power increase, as an expression of stable sustained attention and cognitive control, could simultaneously be involved in auditory disconnection.

The increases we found in functional connectivity between the nodes of the DAN further support the view of an attentional top-down-modulation. The origins of this modulation are probably located in frontal areas (Wang et al., 2016). This would go well with a possible disinhibition of frontal regions (see above) and the correlations of frontal theta and alpha power with the connectivity between frontal (FEF and IFG) and parietal (IPS) nodes of the DAN.

Regarding the DMN, we found connectivity decreases in the posterior part between the medial region of the posterior cingulate cortex (PCC, with adjacent areas of precuneus) and the lateral areas of the left and right inferior parietal lobule (IPL). The DMN as a whole has been associated with functions of self-processing and internal mentation, comprising processes like autobiographical and episodic memory, self-projection into the past and the future, and mind-wandering in general (Northoff et al., 2006; Andrews-Hanna, 2011). The PCC/precuneus complex is a main hub in the structural and functional organization of the brain (Hagmann et al., 2008; Tomasi and Volkow, 2011). This region is a main projection area for the subcortical arousal systems and strongly interconnected with DMN and DAN. Therefore, this hub probably plays a key role in the generation and maintenance of the conscious state (Cavanna and Trimble, 2006). The DMN node comprising the IPL has been implicated in a variety of different functions, among them multisensory integration, bodily self-consciousness, episodic autobiographic memory, and temporal attention (Agosta et al., 2017; Bréchet et al., 2018). The interplay between the medial and lateral parietal nodes of the DMN has been found to be associated with meditative experiences of non-dual awareness (Josipovic, 2014), selflessness (Dor-Ziderman et al., 2013), spacelessness and timelessness (Berkovich-Ohana et al., 2013).

Likewise, findings on psychedelic experiences, which are associated with the dissolution of internal mental activity as well as space, time and self-experience, point to a profound reduction in the activity and connectivity of the posterior DMN (Palhano-Fontes et al., 2015; Smigielski et al., 2019).

Taken together, the disconnections inside the posterior part of the DMN, which we found to be negatively correlated with the increase in frontal theta power may be the main neural correlate of the cessation of internal mentation, as well as the dissolution of self-consciousness, and may also contribute to the experienced state of space- and timelessness. Together, these elements give rise to the phenomenology of CFA (Winter, 2013, 2020).

### Limitations and Conclusion

Our report has certain limitations. (1) It is a single-case study, and from an epistemological point of view, results of single-case studies are always related to the specific person and do not permit generalization. (2) Moreover, the participant studied (TB) possesses unique skills making replication of our results in other participants difficult. (3) The definition of the experimental condition of CFA is not based on an objective physiological marker, but solely based on TB’s report. (4) In this short report the analysis of the EEG is restrained to a limited set of parameters. The presented spectral analysis covers only the most frequently examined frequency bands, theta and alpha. Likewise, analysis of further parameters such as phase synchronization and coherence were not provided.

The single trial reported cannot make specific claims or proof oriented statements. Exploratory single-case studies can, however, complement hypothesis-based confirmatory research and advance science. They can be important sources of new hypotheses and have often disclosed new, hitherto unrecognized phenomena (Bao et al., 2017; Cubelli and Della Sala, 2017). Think for example how much neuroscience has learned from neurological cases such as H.M. or Phineas Gage. Our report may thus contribute to the efforts to narrow down the neural correlates of the conscious state by exploring a rarely experienced state. Future studies should investigate content-reduced and content-free states of consciousness in a more hypothesis-based manner and in more sophisticated and controlled group designs.

Bearing in mind the limitations, we conclude as follows. We report for the first time that the subjectively reported experience of CFA may be associated with a sharp decrease in EEG alpha power as well as a decoupling between the DAN and sensory cortex. This result correlates well with recent findings of decreased alpha power as a marker of sensory disconnection in REM sleep and ketamine anesthesia. We speculate that the extraordinary state of CFA can be classified as a state of disconnected consciousness.

In contrast to REM sleep and ketamine anesthesia, states of CFA lack internal mentation. The reduced connectivity within the posterior DMN may together with the decreased alpha power be a marker of interrupted internal mentation and interrupted self-related processes.

We consider the increase in connectivity within the DAN along with the increase in theta power as a possible signature of a stable attentional state that disconnects the neural correlates of both sensory processes and processes of mental simulation from the neural correlate of consciousness *as such*.

Our findings appear plausible with respect to the reported subjective experience and consistent with a number of previous findings from meditation and consciousness research. Although the results of a single case study are limited, our findings provide a fascinating glimpse at the physiological and neuronal correlates of an extraordinary state which may represent a minimal form of consciousness.

## Data Availability Statement

The datasets generated for this study are available on request to the corresponding author.

## Ethics Statement

The studies involving human participants were reviewed and approved by the Ethics Committee of Albert-Ludwigs University, Albert-Ludwigs University, Freiburg, Germany. The participant provided his written informed consent to participate in this study.

## Author Contributions

UW was responsible for funding acquisition, study conceptualization and study design, the analysis of the EEG, ECG and respiration data, writing the initial draft of the manuscript and manuscript editing, and contributed to data collection and fMRI data analysis. PL conducted data collection, analysis of fMRI data, statistical analysis, and wrote parts of the methods section. TB was the study subject and contributed to the conceptual understanding of the investigated states of consciousness. BA contributed to data collection and fMRI data analysis. MW contributed by reviewing and editing the manuscript and in the ECG analysis. YL assisted in analyzing the EEG data and in drafting the methods section. SS was also responsible for funding acquisition and contributed by supervising the study and reviewing and editing the manuscript. All authors read and finally approved the submitted version.

## Conflict of Interest

The authors declare that the research was conducted in the absence of any commercial or financial relationships that could be construed as a potential conflict of interest.

## Footnotes

1 Similar minimal forms of consciousness may occur as experiences of lucid dreamless sleep (Windt et al., 2016).

2 www.fmrib.ox.ac.uk/fsl
